# A case of ROS1‐rearranged lung adenocarcinoma exhibiting pleural effusion caused by crizotinib

**DOI:** 10.1111/1759-7714.13496

**Published:** 2020-05-20

**Authors:** Hiroaki Tachi, Kengo Nishino, Taisuke Nakaizumi, Kenya Kuramoto, Kei Shimizu, Yusuke Yamamoto, Keisuke Kobayashi, Hideo Ichimura, Akiko Sakata, Takeshi Nawa

**Affiliations:** ^1^ Department of Respiratory Medicine Hitachi General Hospital, Hitachi Ltd. Hitachi City Japan; ^2^ Department of Thoracic Surgery Hitachi General Hospital, Hitachi Ltd. Hitachi City Japan; ^3^ Department of Pathology Hitachi General Hospital, Hitachi Ltd. Hitachi City Japan

**Keywords:** Complete response, crizotinib, lung adenocarcinoma, pleural effusion, ROS1 rearrangement

## Abstract

Reports of crizotinib‐induced pleural effusion in non‐small cell lung cancer (NSCLC) are limited. A 35‐year‐old Japanese woman was diagnosed with ROS1‐rearranged lung adenocarcinoma (primary left lower lobe, cT4N3M1c). Crizotinib was administered as first‐line therapy, and the primary and mediastinal hilar lymph node metastases rapidly shrank. On the fourth day of treatment, chest X‐ray demonstrated contralateral pleural effusion. On the 41st day of treatment, crizotinib was discontinued because of grade 3 neutropenia. Examination including surgical thoracoscopy did not reveal causative findings, and the continued cessation of drug administration enabled the right pleural effusion to decrease gradually and disappear, suggesting that this event was a side effect of crizotinib. The disease did not progress even though the drug was withdrawn for more than one year. In conclusion, crizotinib was considered to cause pleural effusion as an adverse event in a case of ROS1‐rearranged lung adenocarcinoma with a complete response.

## Introduction

ROS1 rearrangement has been estimated to be present in 1% to 2% of patients with non‐small cell lung cancer (NSCLC).^1^, ^2^ Crizotinib, an inhibitor of anaplastic lymphoma kinase (ALK), is known to have marked antitumor activity in patients with ROS1‐positive advanced NSCLC^3^ because ROS1 is considered to have a high homology with the tyrosine kinase region of ALK due to its protein structure.^4^

Pleural disorder is one of the clinical phenotypes of drug‐induced lung injury. Although pleural effusion and pleurisy are listed as adverse events for many drugs, they are rarely observed in clinical practice. This report describes a case of ROS1‐rearranged lung adenocarcinoma exhibiting contralateral pleural effusion caused by crizotinib.

## Case report

**Figure 1:**
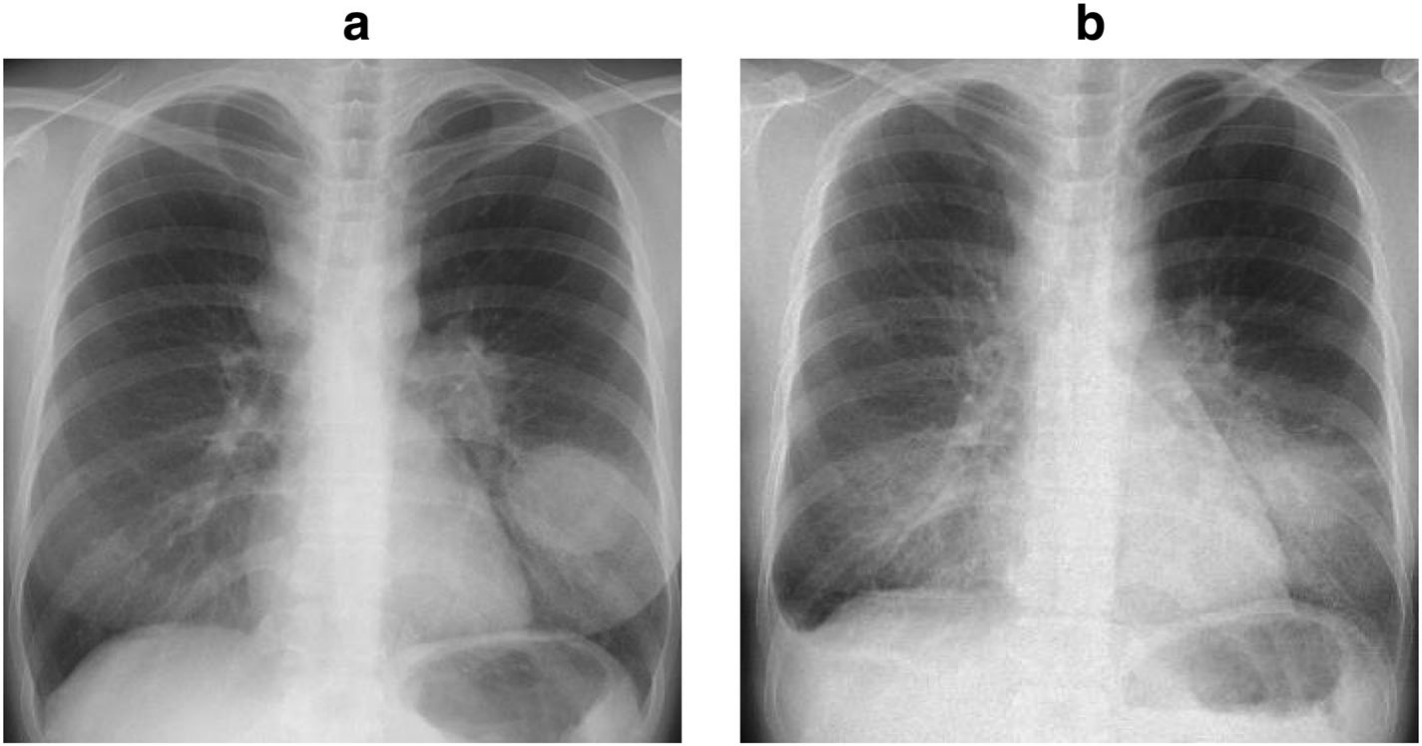
Chest X‐ray findings. (**a**) Pretreatment. A large mass shadow was observed in the left lower lung field, and enlarged lymph nodes were found in the left hilum and right mediastinum. (**b**) Day 4 of treatment. Right pleural effusion and ground‐glass appearance of the bilateral lungs distributed dominantly on the side of the hilum were observed.

**Figure 2:**
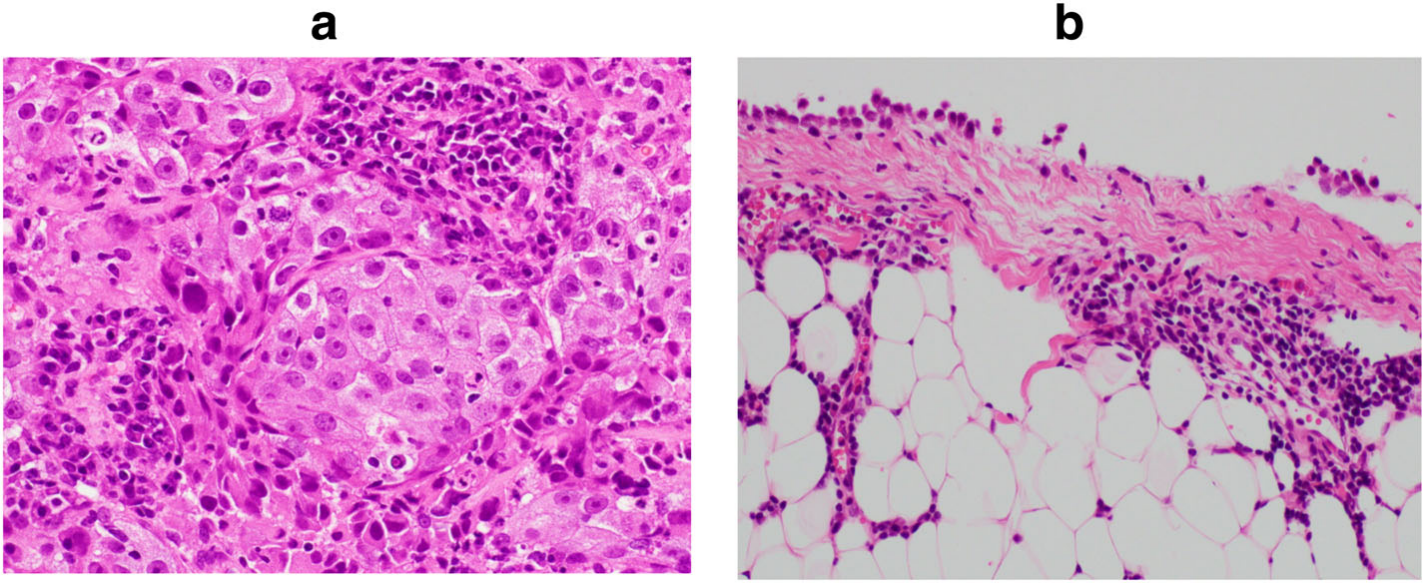
Histopathological findings. (a) Bronchial biopsy findings from the mass in the left lower lobe (HE staining ×400). The tumor grew solidly without glandular structure, being composed of neoplastic cells with irregularly enlarged and strongly atypical nuclei. (b) Parietal pleural biopsy findings (HE staining ×200). Only lymphocytes, plasma cells, and reactive mesothelial cells were found, and there was no malignancy.

A 35‐year‐old Japanese woman was referred to our hospital for evaluation of a mass in the left lower lung field (Fig 1a) with a complaint of dry cough for six months. She had a smoking history of 15 pack‐years but no notable past medical history or drug allergy. Chest computed tomography demonstrated a large mass in the left lower lobe of her lung, and enlarged lymph nodes in the left hilum and right mediastinum. Solid adenocarcinoma was detected by bronchial biopsy from the mass in the left lower lobe (Fig 2a). The cancer stage was determined to be cT4N3M1c, stage IVB, isolated right cervical lymph node metastasis. Molecular testing of the biopsied specimen revealed ROS1 rearrangement.

**Figure 3:**
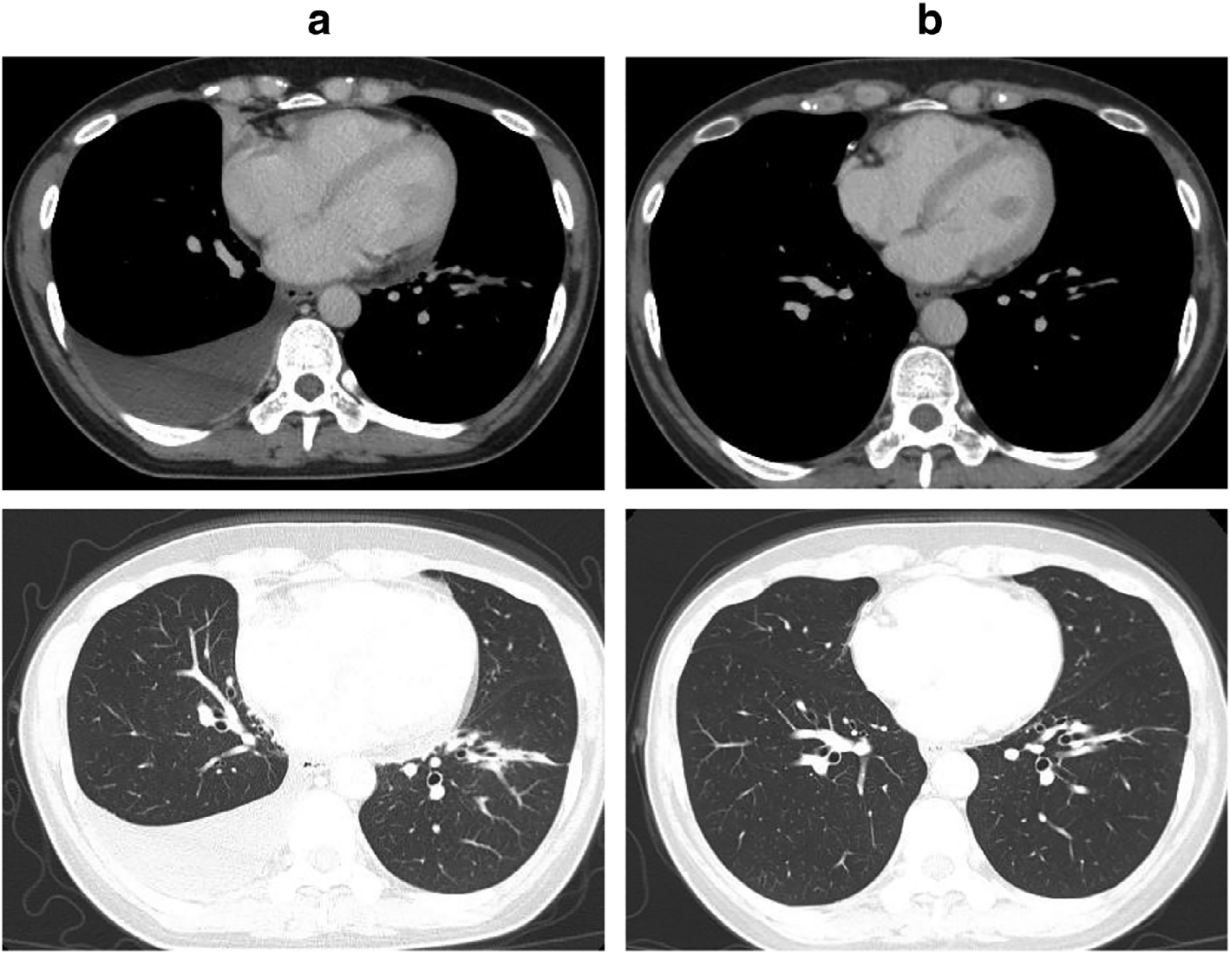
Chest computed tomography (CT) findings. (a) Eight weeks after starting treatment although the large mass shadow significantly disappeared, right pleural effusion was observed without pleural dissemination nodules. (b) One year after withdrawal and the right pleural effusion gradually decreased and disappeared. Moreover, no regrowth of the primary lesion was observed.

Crizotinib was introduced as the first‐line therapy (250 mg twice daily). The primary lesion and mediastinal hilar lymph node metastases both shrank rapidly. However, right pleural effusion was observed on chest X‐ray on the fourth day of treatment (Fig 1b). The right pleural effusion was exudative and predominantly composed of lymphocytes, but cytology and culture were both negative (Table 1). For autoimmune markers, only antinuclear antibody and anti‐ds‐DNA IgG were measured, both of which were negative. Cardiac ultrasonography demonstrated normal cardiac function and no evidence of heart failure. During crizotinib administration, right pleural effusion continued to increase, but after 41 days of treatment, crizotinib was discontinued due to grade 3 neutropenia, followed by a gradual decrease in pleural effusion. Surgical thoracoscopy was performed one month after the cessation of crizotinib. There were no causative findings of pleural effusion in the right pleura within the visible range. Biopsy of the parietal pleura and partial resection of the collapsed right middle lobe were performed. On pathology, there were no malignant findings. Lymphocytes, plasma cells, and reactive mesothelial cells were observed (Fig. 2b). As right pleural effusion disappeared and did not recur during continued drug withdrawal, it was considered to be an adverse event due to crizotinib. Even without medication for more than one year, both the primary lesion and mediastinal hilar lymph node metastases disappeared, and no new lesions developed (Fig 3).

**Table 1:** Laboratory findings (blood test and pleural fluid analysis)

Blood test								Pleural fluid analysis		
CBC				Serum chemistry				Color	Pale yellow	
WBC	116	×10^2^/μL		TP	7.1	g/dL		S.G.	1.025	
Neu	80	%		ALB	3.8	g/dL		Cells	5676	/μL
Eos	4	%		AST	18	U/L			(only lymphocyte)	
Bas	0	%		ALT	18	U/L		Protein	3.5	g/dL
Mono	5	%		LDH	345	U/L		LDH	127	U/L
Lym	11	%		ALP	189	U/L		Glucose	106	mg/dL
RBC	444	×10^4^/μL		T‐Bil	0.3	mg/dL		ADH	11.3	
Hb	12.7	g/dL		BUN	10.8	mg/dL		Culture	negative	
PLT	30.8	×10^4^/μL		Cre	0.66	mg/dl		Cytology	negative	
				Na	141	mmol/L				
Tumor marker				K	4.3	mmol/L				
CEA	3.8	ng/mL		Cl	105	mmol/L				
CYFRA	4.8	ng/mL		Ca	8.5	mg/dL				
PRO GRP	43.8	pg/mL		CRP	0.6	mg/dL				
SLX	34.0	U/mL		BNP	<5.80	pg/mL				
SCC	53.0	ng/mL								
NSE	25.0	ng/mL		Autoimmume marker						
				Antinuclear antibody		<40				
				Anti ds‐DNA IgG		<10				

## Discussion

To the best of our knowledge, this is the first report of ROS1‐rearranged lung adenocarcinoma exhibiting pleural effusion caused by crizotinib with a complete response.

A diffuse alveolar damage (DAD) pattern^5^, ^6^, ^7^, ^8^ and hypersensitivity pneumonia pattern^7^, ^9^ have been reported as lung adverse events caused by crizotinib for lung adenocarcinoma with ALK rearrangement. However, we found no case reports describing noncardiogenic pleural effusion due to crizotinib.

Crizotinib has inhibitory activity against CYP3A4 and may increase the blood concentration of other drugs. In this case, although the patient had been taking other medications (eg, morphine sulfate hydrate, acetaminophen, celecoxib, esomeprazole magnesium hydrate, metoclopramide, magnesium oxide, and levocetirizine hydrochloride), it is unlikely that they were the causative agents because there have been no reports of pleural effusion caused by these drugs and none whose metabolism is completely dependent on CYP3A4 were included. As the pleural effusion decreased and disappeared after the cessation of crizotinib, it was thought to be the causative agent. However, the involvement of concomitant medications was unable to be excluded because the pleural effusion began to decrease after withdrawing crizotinib and these drugs were subsequently discontinued as the patient's condition improved.

Regarding the mechanism of pleural effusion, Gemma *et al*.^8^ previously reported that crizotinib‐induced lung injury with pulmonary edema‐like shadows may be accompanied by bilateral pleural effusion. In our case, a ground‐glass appearance of the bilateral lungs distributed dominantly in the hilum was observed on chest X‐ray on the fourth day of treatment, but it was difficult to consider it to be the same mechanism because a similar shadow, suggesting carcinomatous lymphangiomatosis, was noted the day before starting treatment.

In addition, a stable course without recurrence for more than one year after the discontinuation of crizotinib is considered to be markedly rare. According to a previous clinical study,^3^ the complete response rate was 6%. On the other hand, a Japanese study^10^ reported that the objective response rate of ROS1‐rearranged NSCLC to crizotinib was 80%. Crizotinib was reported to bind significantly more strongly to ROS1 than to ALK,^11^ which may lead to effective target suppression and lasting therapeutic effects. One basic study suggested that the combination of cisplatin and high‐dose crizotinib induces immunogenic cell death in NSCLC.^12^

In conclusion, crizotinib was considered to cause pleural effusion as an adverse event in a patient with ROS1‐rearranged lung adenocarcinoma.

## Disclosure

The authors have no conflicts of interest to declare.
